# Simple and inexpensive three-step rapid amplification of cDNA 5′ ends using 5′ phosphorylated primers

**DOI:** 10.1016/j.ab.2012.10.031

**Published:** 2013-03-01

**Authors:** Kai Dallmeier, Johan Neyts

**Affiliations:** Rega Institute for Medical Research, Department of Virology and Chemotherapy, KULeuven–University of Leuven, B-3000 Leuven, Belgium

**Keywords:** Transcriptional initiation site mapping, Rapid amplification of cDNA ends (RACE), RNA cloning, Linker cloning, Tobacco acid pyrophosphatase

## Abstract

Rapid amplification of cDNA 5′ ends (5′-RACE) is routinely used for the sequence analysis of the upstream noncoding regions of cellular mRNAs; however, it represents a tedious and cost-intensive procedure. By employing 5′ phosphorylated gene-specific primers for first-strand cDNA synthesis, we cut short the previously established reverse ligation and amplification protocol of Mandl and coworkers (BioTechniques, 1991, vol. 10, pp. 484–486) to a streamlined three-step procedure that no longer depends on enzymatic mRNA decapping or linker ligation. The novel three-step protocol has been validated by mapping the transcriptional start sites of heterologously expressed yellow fever virus genomic RNAs from cultured mammalian cells.

Determining the sequence of RNAs is a widely used routine in molecular biology, to which end mostly reverse transcription and consecutive polymerase chain reaction (PCR)^1^ is employed. However, sequencing the utmost 5′ ends of a specific RNA still remains a tedious and fairly costly procedure (see Ref. [1] and references therein for a critical review). Here the method of choice is the so-called rapid amplification of cDNA ends (RACE) originally introduced in Ref. [2]. RACE procedures are commercially available in several customized versions in a kit format, mainly based on the strategies described in Refs. [3–5]. In general, rapid amplification of cDNA 5′ ends (5′-RACE) for an eukaryotic mRNA is a multistep procedure that includes (i) removal of the 5′ 7-methyl-guanosine (7mGppp) cap by tobacco acid pyrophosphatase (TAP), (ii) ligation of an oligoribonucleotide linker to the resulting 5′-phosphate by the activity of the phage T4 RNA ligase 1 (RNL1), (iii) reverse transcription by an RNA-dependent DNA polymerase (reverse transcriptase, RT) that uses as a primer an oligodesoxynucleotide (ODN) complementary to the synthetic RNA linker added in step (ii) and (iv) subsequent PCR on this first-strand cDNA with the linker-specific primer already used in step (iii) and a second gene-specific reverse primer. Finally, these PCR products are cloned in appropriate plasmid vectors for sequence analysis. A method introduced by Mandl and coworkers [6] for the simultaneous cloning of the RNA 5′ and 3′ ends needs to be considered as a variant of this general four-step protocol, but substituting ligation of a synthetic RNA linker (step ii) by an intramolecular ligation (RNA circularization) prior to subsequent RT–PCR using a pair of inverted gene-specific ODN primers (Fig. 1A).

Here we present a further improvement to the method in terms of simplicity and reduced costs that is especially suited for 5′-RACE. The novel streamlined three-step 5′-RACE employs the following steps: (i) reverse transcription with 5′ phosphorylated gene-specific primers, (ii) cDNA circularization by RNL1 and (iii) PCR amplification using inverted primers followed by blunt end cloning for sequencing. As a key innovation, the RNA of interest is reverse-transcribed directly during the first step. By this means, a 5′ phosphorlyated end is introduced into the nascent cDNA (resulting from elongation of ODN 1), relieving from any further enzymatic RNA manipulation and/or synthetic RNA linker ligation (Fig. 1B). This first-strand cDNA is directly circularized by use of T4 RNL1 and is finally PCR amplified with inverted primers ODN 2 and ODN 3. The resulting amplicon covers the junction between the original RNA 5′ end joined to the reverse complement of ODN 1 (Fig. 2).

For a proof-of-concept, we amplified the 5′ ends of yellow fever virus vaccine strain 17D (YFV-17D, GenBank X03700.1) that was cloned under transcriptional control of the Simian virus 40 (SV40) genomic origin/promoter (pSV40–YF17D, K. Dallmeier, unpublished) to assess proper transcriptional start site selection [7,8] following plasmid DNA transfection into Vero-B (African green monkey kidney) cells as needed for productive viral RNA replication [9]. To that end, 0.5 μg of DNase-I-treated total cellular RNA (RNeasy, Qiagen) was reverse-transcribed by 5 U of Moloney murine leukemia virus (M-MLV) RT (Promega) using 50 pmol of primer YF17D(–)180/*Eco*RV-5′P (5′-phospho-GATATCCGAACTCCTCGTCGTACC-3′, where *Eco*RV recognition site is underlined) in a total volume of 12.5 μl at 42 °C in the presence of 10 U RNasin RNase inhibitor (Promega). Reactions were terminated after 30 min by heating to 95 °C for 15 min and subsequently adding 3 volumes of TE buffer (10 mM Tris–HCl and 1 mM ethylenediaminetetraacetic acid [EDTA], pH 8.0) supplemented with 4 μg ml^−1^ RNase A (Promega). Second, two-fifths of the resulting cDNAs was circularized by 20 U of T4 RNL1 (New England Biolabs) in the presence of 15% (w/v) polyethylene glycol (PEG) 8000 (Sigma–Aldrich) in a total volume of 50 μL at 37 °C for 60 min. Unreacted residual first-strand primers and cDNAs were removed by the addition of 1.5 U of T4 DNA polymerase for 30 min at 37 °C employing its 3′ → 5′ exonuclease activity. Third, 2 μl of these intramolecularly joined 5′ cDNAs was amplified by 35 cycles of PCR (KAPA HiFi Hotstart ReadyMix, KAPA Biosystems) using inverted primer pairs, namely for YFV-17D primers YF17D(–)90 (5′-ACGAACGATTAAAATTAATCCA-3′) plus YF17D(+)120 (5′-TGTCTGGTCGTAAAGCTCA-3′). Finally, amplicons of expected size (157 bp) could be observed by 2% Tris–acetate–EDTA (TAE) agarose gel electrophoresis, purified from the gels (QIAquick, Qiagen), and cloned into pJET1.2/blunt (CloneJET PCR Cloning Kit, Fermentas). Of note, amplicons could be observed only if both reactions were carried out, reverse transcription with ODN 1 YF17D(–)180/*Eco*RV-5′P and cDNA circularization by RNL1, but not in control reactions omitting either or both enzymes prior to inverted RT–PCR (not shown). The amplicon was visible as a single DNA species without multiple or unspecific side bands, much in contrast to what others have witnessed as a major drawback of inverted PCR on larger cDNAs (several kilobases) directly following circularization [10]. Plasmid DNA minipreps of several clones were subjected to Sanger sequencing (BigDye 3.1, Applied Biosystems).

To access how accurately the ultimate 5′ ends could be amplified by the novel three-step approach, RNA from supernatant of YFV-17D infected Vero-B cells representing 5′ 7mGppp cap infectious full-length viral genomic RNA was included as control. Prior to the three-step 5′-RACE, the control RNA was consecutively pretreated with thermosensitive alkaline phosphatase (TSAP, Promega), 5′-monophosphorylated by phage T4 polynucleotide kinase (PNK, New England Biolabs), and finally subjected to digestion by the yeast 5′ → 3′ exoribonuclease 1 (Xrn-1, New England Biolabs). This treatment should render any noncapped RNA species sensitive to exonucleolytic degradation by Xrn-1 [11]. Indeed, viral RNA from the supernatant of YFV-17D infected cells remained nuclease resistant under these circumstances, whereas a synthetic YFV-17D RNA generated by in vitro transcription (Ribomax Sp6 RNA Production System, Promega) of a cloned YFV-17D cDNA present in pACNR-FLYF17DII [12] (kindly provided by P. Bredenbeek, Leiden UMC, Leiden, The Netherlands) became Xrn-1 sensitive on this stepwise enzymatic conversion of the nascent Sp6 RNA polymerase triphosphorylated transcript 5′ ends into 5′ monophosphates [11] (not shown).

By this novel three-step 5′-RACE approach, the transcriptional start site of the heterologously expressed YFV-17D genomic RNA (pSV40–YF17D) could be mapped to one of two equally frequently occurring locations: (i) the natural YFV-17D start adenine–guanosine dinucleotide (+1 and +2 in Fig. 2) (5 of 25 clones) immediately downstream of the artificially fused pyrimidine-rich upstream sequence of the SV40 promoter [7,8] or (ii) the second guanosine residue (+2) (8 of 25 clones). Such an offset by one nucleotide is in line with a somewhat imprecise start site selection reported for the SV40 promoter/origin [7,8]. Sequencing of the natural YFV-17D RNA revealed similar results, yet (fully as expected) without any skew toward starting from nucleotide position +2 (not shown).

The method showed reasonable precision, with the 5′ ends of 14 out of 25 clones analyzed mapped to either of these two major initiation sites. In fact, only one incidental 5′-terminal nucleotide addition could be detected during our study (Fig. 2, clone 2), translating into a relatively low incidence of less than 5% of this kind of an error. It can probably be attributed to the intrinsic terminal nucleotidyl transferase activity associated with RTs in general [13–16] rather than reverse transcription of the terminal 5′–5′ linked 7mGppp cap nucleotide [17] because it represented a C residue rather than a G residue. A minor bias toward slightly shorter transcripts (Fig. 2, clones 2, 6, 7, 11, 17, 18, and 22) was possibly due to unwanted exonucleolytic processing of the target RNA and ODN 1 linker during RNA extraction and successive enzymatic manipulation. Moreover, an additional four clones containing reasonably larger 5′ deletions of 45, 52, 121, and 139 bases were detected (not shown in figure). The relatively high frequency of variants representing terminal 5′ deletions (10 of 25 clones) may be explained by an overall poor RNA quality resulting from YFV-17D-induced cell death [12]. In fact, the total RNA of the transfected cells used as starting material for the three-step 5′-RACE already showed reasonable degradation, as visualized by ethidium bromide staining following agarose gel electrophoresis (not shown), all the more corroborating the sensitivity and robustness of the approach.

Of note, among the 25 clones analyzed, a total of 12 different sequence variants were present, resulting in a (formally calculated) high frequency of independent cDNA clones of roughly every second molecule analyzed. In line with this, the occurrence of the C1A variant (cDNA clone 2) can be readily explained by the terminal transferase activity of the M-MLV RT preceding the amplifying PCR step (see Discussion above) and not by base incorporation errors during PCR, as expected for a massive amplification preceding cloning. Taken together, the skew toward frequent variants most likely represents the expected (and relevant) major transcriptional start sites of the SV40 promoter used, as opposed to PCR duplicates holding no additional information.

In summary, the novel three-step 5′-RACE can fully substitute for the more tedious and costly protocols currently available by delivering comparable precision and reliability regarding the sequence information obtained. A possible disadvantage of our novel protocol is a lack in specificity for the 7mGppp cap (originally conferred by the activity of TAP) during the first cloning step [3,6], although this is of relevance only with canonically capped eukaryotic mRNAs. In fact, cloning by the new three-step protocol will not discriminate against 5′ capped and 5′ non-, mono-, di-, or triphosphorylated RNA species, allowing the picking up of any RNA (including endo- and exonucleolytically processed RNAs and prokaryotic RNAs; see below). Nevertheless, comparable specificities should be readily achievable by predigestion of the total input RNA by appropriate exonucleases, such as pretreatment with Xrn-1 to enrich for capped RNA [11], as demonstrated by the use of the in vitro transcribed synthetic cognate RNA (see above).

The main advantages of the method presented here are (i) its robustness and (ii) its target RNA specificity, possibly due to the early conversion of the target RNA to cDNA without the further need of specialized enzymatic manipulation and the use of three (rather than one [2,18] or two [6,10,19]) gene-specific primers. The three-step protocol can be performed without using any expensive kits, RNA linkers, or TAP—just readily available standard DNA/RNA modifying enzymes and simple ODN primers. We especially emphasize that our approach might in particular be an easy-to-perform (and highly cost-effective) alternative to any elaborate, possibly second-choice approach fitting more specialized demands. This may include the need for further amplification of rare transcripts by nested PCR or rolling circle amplification [10], to achieve higher sensitivities and specificities, or special demands in connection with the cloning of cDNA libraries or the analysis of both RNA 5′ and 3′ ends at the same time [1,6,10]. Of note, a fairly similar approach to our three-step protocol for the mapping of transcriptional start sites in bacteria was published very recently [20] during the drafting of this manuscript, proving the general applicability of the approach presented.

## Figures and Tables

**Fig.1:**
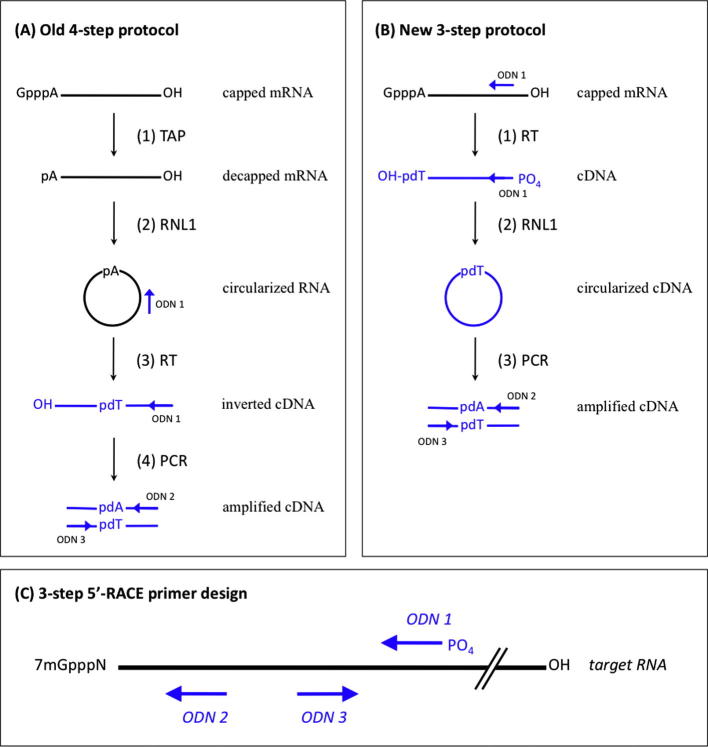
Principal layout of improved three-step version of 5′-RACE (B) in comparison with the established circularization/reverse amplification approach (A). For a detailed explanation, see text. (C) Principal layout for primer design of three-step 5′-RACE. TAP, tobacco acid phosphatase; RNL1, T4 RNA ligase 1; ODN, oligodesoxynucleotide; RT, reverse transcriptase; PCR, polymerase chain reaction.

**Fig.2:**
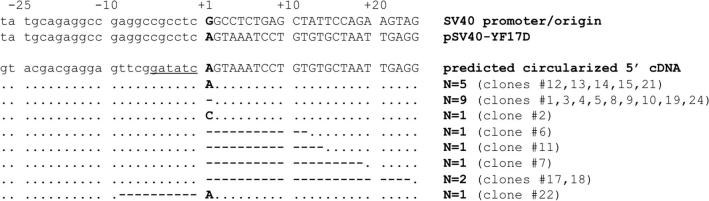
Sequence analysis of heterologously expressed YFV-17D RNA by three-step 5′-RACE. (A) Sequences of cDNA clones from pSV40–YF17D transfected cells were aligned with the predicted circularized 5′ cDNA as expected progeny from the SV40 origin/promoter fusion to the YFV-17D cDNA in pSV40–YF17D (second line). The predicted major transcriptional start site for the SV40 promoter/origin (+1, top line) according to Refs. [7,8] is shown. The *Eco*RV recognition site derived from the 5′ end of ODN 1 is underlined. Dots represent the consensus with the predicted sequence, and dashes represent nucleotide deletions.

## Footnotes

1 *Abbreviations used:* PCR, polymerase chain reaction; cDNA, complementary DNA; RACE, rapid amplification of cDNA ends; 5′-RACE, rapid amplification of cDNA 5′ ends; mRNA, messenger RNA; 7mGppp, 7-methyl-guanosine; TAP, tobacco acid pyrophosphatase; RNL1, T4 RNA ligase 1; RT, reverse transcriptase; ODN, oligodesoxynucleotide; EDTA, ethylenediaminetetraacetic acid; YFV-17D, yellow fever virus vaccine strain 17D; SV40, Simian virus 40; M-MLV, Moloney murine leukemia virus; Xrn-1, exoribonuclease 1.
